# Physiology and pathology of eosinophils: Recent developments: Summary of the Focus Workshop Organized by DGAKI

**DOI:** 10.1111/sji.13032

**Published:** 2021-03-11

**Authors:** Harald Renz, Claus Bachert, Claudia Berek, Eckard Hamelmann, Francesca Levi‐Schaffer, Ulrike Raap, Hans‐Uwe Simon, Sabine Ploetz, Christian Taube, Peter Valent, David Voehringer, Thomas Werfel, Nan Zhang, Johannes Ring

**Affiliations:** ^1^ Institute of Laboratory Medicine Universities of Giessen and Marburg Lung Center (UGMLC) German Center for Lung Research (DZL) Philipps Universität Marburg Marburg Germany; ^2^ Upper Airways Research Laboratory and Department of Oto‐Rhino‐Laryngology Ghent University and Ghent University Hospital Ghent Belgium; ^3^ Division of ENT Diseases CLINTEC Karolinska Institute University of Stockholm Stockholm Sweden; ^4^ Deutsches Rheuma Forschungszentrum Ein Institut der Leibnizgemeinschaft Berlin Germany; ^5^ Klinik für Kinder‐ und Jugendmedizin Evangelisches Klinikum Bethel Bielefeld Germany; ^6^ Allergy Center of the Ruhr University Bochum Germany; ^7^ School of Pharmacy Faculty of Medicine The Institute for Drug Research The Hebrew University of Jerusalem Israel; ^8^ Clinics of Dermatology and Allergy Faculty of Medical Health and Sciences University of Oldenburg Germany; ^9^ Institute of Pharmacology University of Bern Bern Switzerland; ^10^ Skin Center Harlaching Munich Germany; ^11^ Department of Pulmonary Medicine University Hospital Essen—Ruhrlandklinik Essen Germany; ^12^ Department of Internal Medicine I Division of Hematology and Hemostaseology, and Ludwig Boltzmann Institute for Hematology & Oncology Medical University of Vienna Vienna Austria; ^13^ Department of Infection Biology University Hospital Erlangen and Friedrich‐Alexander University Erlangen‐Nuremberg Erlangen Germany; ^14^ Klinik für Dermatologie Allergologie und Venerologie Medizinische Hochschule Hannover Hannover Germany; ^15^ Deptment of Dermatology and Allergology Biederstein Technical University Munich (TUM) Munich Germany

**Keywords:** anti‐interleukin 5, DNAzyme against GATA3; eosinophil, eosinophil diseases, hypereosinophilic syndrome

## Abstract

Over the last century, eosinophils have been regarded ambiguously either as ‘friends’ or ‘foes’. Recent developments have greatly enhanced our understanding of the role and function of eosinophils in health and disease. Pathogenic eosinophilic inflammation can lead to severe diseases in various organs, such as the gastrointestinal tract, airways, heart and skin. In a 2‐day focus workshop of the German Society for Allergology and Clinical Immunology (DGAKI), the state of the art was discussed and practical recommendations for diagnosis and treatment of eosinophilic diseases, with a particular focus on new biologics, such as anti‐interleukin 5 and anti‐interleukin 5R, were derived.

**Abbreviations**
AEU: Allergic effector unit
APRIL: A proliferation‐inducing ligand
CCR: Chemokine receptor
CRS: Chronic rhinosinusitis
Dsg: Desmoglein
ECP: Eosinophil cationic protein
EET: Extracellular eosinophilic trap
EGID: Eosinophilic gastrointestinal diseases
Egpa: Eosinophilic granulomatosis and polyangiitis
EoE: Eosinophilic esophagitis
Eos: Eosinophils
FDA: Food and Drug Administration
GI: Gastro‐intestinal
GM‐CSF: Granulocyte Monocyte colony‐stimulating factor
HE: Hypereosinophilia
HES: Hypereosinophilic syndrome
IgA: Immunoglobulin A
IL‐33: Interleukin 33
JAK2: Janus kinase 2
LPS: Lipopolysaccharide
mAbs: Monoclonal antibodies
MBP: Major basic protein
MCs: Mast cells
MLKL: Mixed lineage kinase‐like
PDGFRA: Platelet‐Derived Growth Factor receptor alpha
PPI: Proton pump inhibitors
RIPK3: Receptor‐interacting protein kinase 3
SEA‐SEE: Staphylococcal enterotoxin A‒E
SIGLEC‐F: Sialic acid‐binding immunoglobulin‐type lectins
SplD: *Staphylococcus aureus*‐derived protein
Th: T helper cell
TSLP: Thymic stromal lymphopoietin
TSST‐1: Toxic shock syndrome toxin‐1
WHO: World Health Organization

## 1 INTRODUCTION

Eosinophilic granulocytes are among the most distinctive cells in human blood, mainly due to the bright colour given by eosin, an aniline dye first used in 1877 by Paul Ehrlich^1^ for the differentiation of white blood cells. In subsequent decades, there was marked controversy over whether these cells could be regarded as ‘good’ or ‘bad’,^2^ and lately there has been marked progress and increasing interest in these cells. Therefore, the German Society of Allergology and Clinical Immunology (DGAKI) organized a ‘Focus’ workshop to reflect the state of the art in this field and discuss practical aspects of management of eosinophilic diseases. Here, we summarize the outcomes of this forum, focussing on current developments, and discuss clinical and experimental research of note in the field of eosinophil granulocytes and eosinophil‐related diseases.

## 2 BIOLOGY OF EOSINOPHILS

**FIGURE 1:**
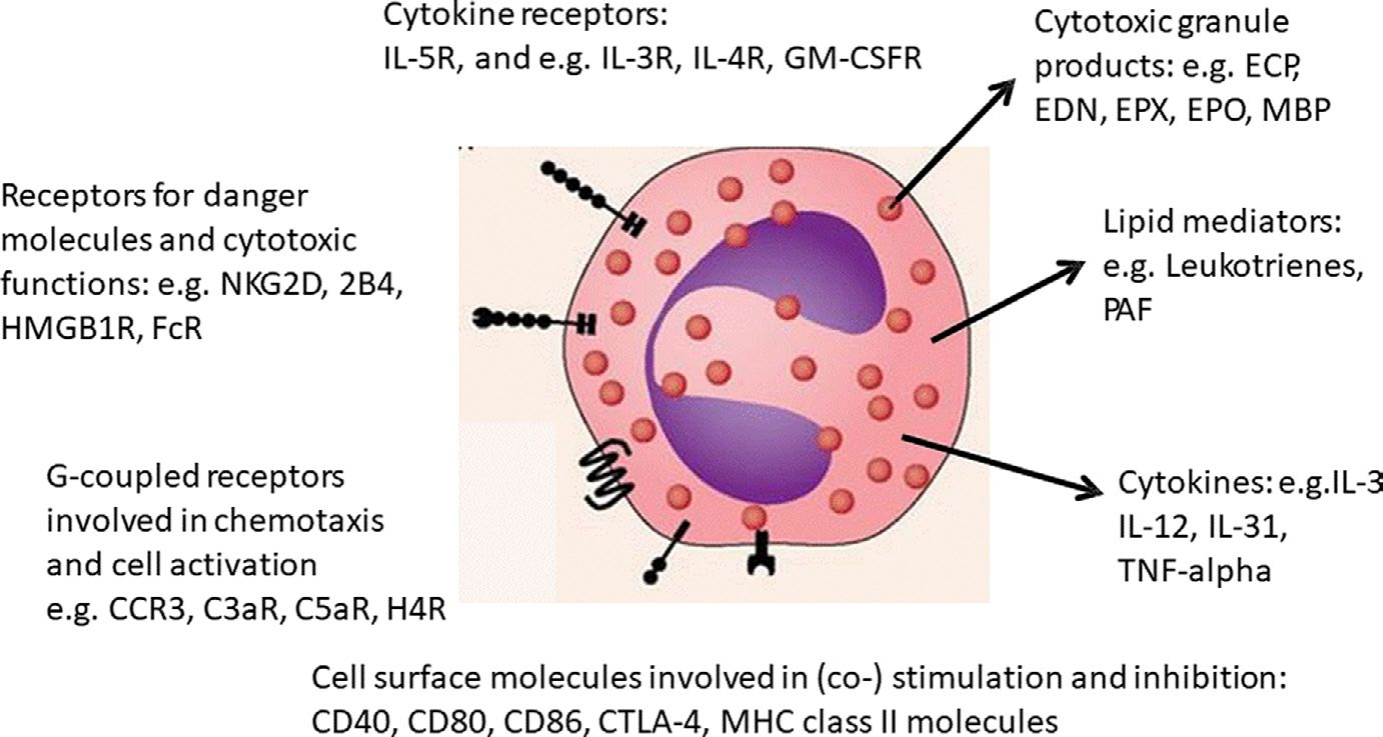
Examples of eosinophilic mediators and receptors involved in allergic inflammation

The pro‐inflammatory effects of eosinophils, exerted through release of toxic mediators, cytokines and other products and causing symptoms of allergic disorders (Figure 1), have generated much attention. However, eosinophils have many physiological functions.^3^, ^4^ Eosinophils are a major cell population of the gastrointestinal (GI) tract, and their homing to the lamina propria is independent of inflammation. Indeed, the first eosinophils appear in the GI tract already during foetal development, long before the gut is colonized by the microbiota. The development of normal‐sized Peyer´s patches in the GI tract requires eosinophils, and in their absence, IgA class switching is impaired.^5^ Consequently, intestinal and serum IgA is significantly reduced in eosinophil‐deficient mice. Furthermore, eosinophils play a crucial role in immune homeostasis, as they control T cell responses to environmental antigens in mucosal tissues of the lung and the GI tract,^6^ and prevent inflammatory reactions induced by infections with natural parasites, such as the bacterium *Helicobacter pylori*.^7^ Eosinophils also have an essential role in long‐term immune protection. In the bone marrow, they provide the cytokine APRIL, which promotes plasma cell longevity.^8^ Furthermore, eosinophils play an important role during puberty,^9^ in the structural development of adipose tissue, and in tumour and transplant rejection.^2^ Although hypereosinophilia in relation to tumours was first described more than 120 years ago, its role in tumours is still undefined.^10^ Eosinophils release antitumorigenic (eg TNF‐α, cationic proteins and IL‐18) and protumorigenic molecules (eg pro‐angiogenetic factors) depending on the tumour microenvironment.^11^, ^12^ In several neoplasias (eg melanoma, gastric, colorectal, oral and prostate cancer), eosinophilia was related to a better outcome, whereas in others, such as Hodgkin's lymphoma, eosinophilia has been linked to poor prognosis.^12^, ^13^ A better understanding of the paradoxical role in cancer progression could help design therapeutic strategies.

Eosinophils are components in adipose tissue infiltrates and promote glucose homeostasis.^14^ Mesenchyme‐derived stromal cells in white adipose tissue activate resident type 2 innate lymphoid cells (ILC2) to release IL‐5.^15^ IL‐5 activated eosinophils release IL‐4, which, together with ILC2 released IL‐13, induces the conversion of resident tissue macrophages to activated macrophages able to release norepinephrine‐stimulating adipose browning.^16^, ^17^ Eosinophil‐deficient mice developed weight gain and glucose intolerance whereas hypereosinophilic mice displayed neither weight gain and nor glucose intolerance when fed a high‐calorie diet.^14^ However, recent data suggest that elevating adipose eosinophils in obese mice by IL‐5 did not rescue metabolic impairments.^18^ Further research needs to clarify these findings. Transgenic mouse models have allowed new understanding of eosinophil functions. The IL‐4eGFP reporter mice were used to identify eosinophil‐committed precursor cells in the foetal liver where they express low levels of Siglec‐F, and no CCR3.^19^ In adult mice, resting and activated eosinophils can be distinguished by expression of CD62L, Siglec‐F, Pir‐B and CD29 on the cell surface.^19^

Corresponding functional studies have provided new insights into the physiology of eosinophils. Interestingly, analysis of eosinophil turnover by means of BrdU incorporation assays has revealed that helminth‐induced tissue eosinophilia was caused by recruitment of already existing eosinophils, rather than that of de novo‐generated eosinophils from the bone marrow.^20^ Mice with eosinophil‐specific expression of the Cre recombinase have demonstrated an important role of the NF‐kappa B pathway for regulation of eosinophilia after helminth infection.^21^ Furthermore, the alarmin IL‐33 can promote survival of eosinophils by autocrine GM‐CSF signalling.^22^

**FIGURE 2:**
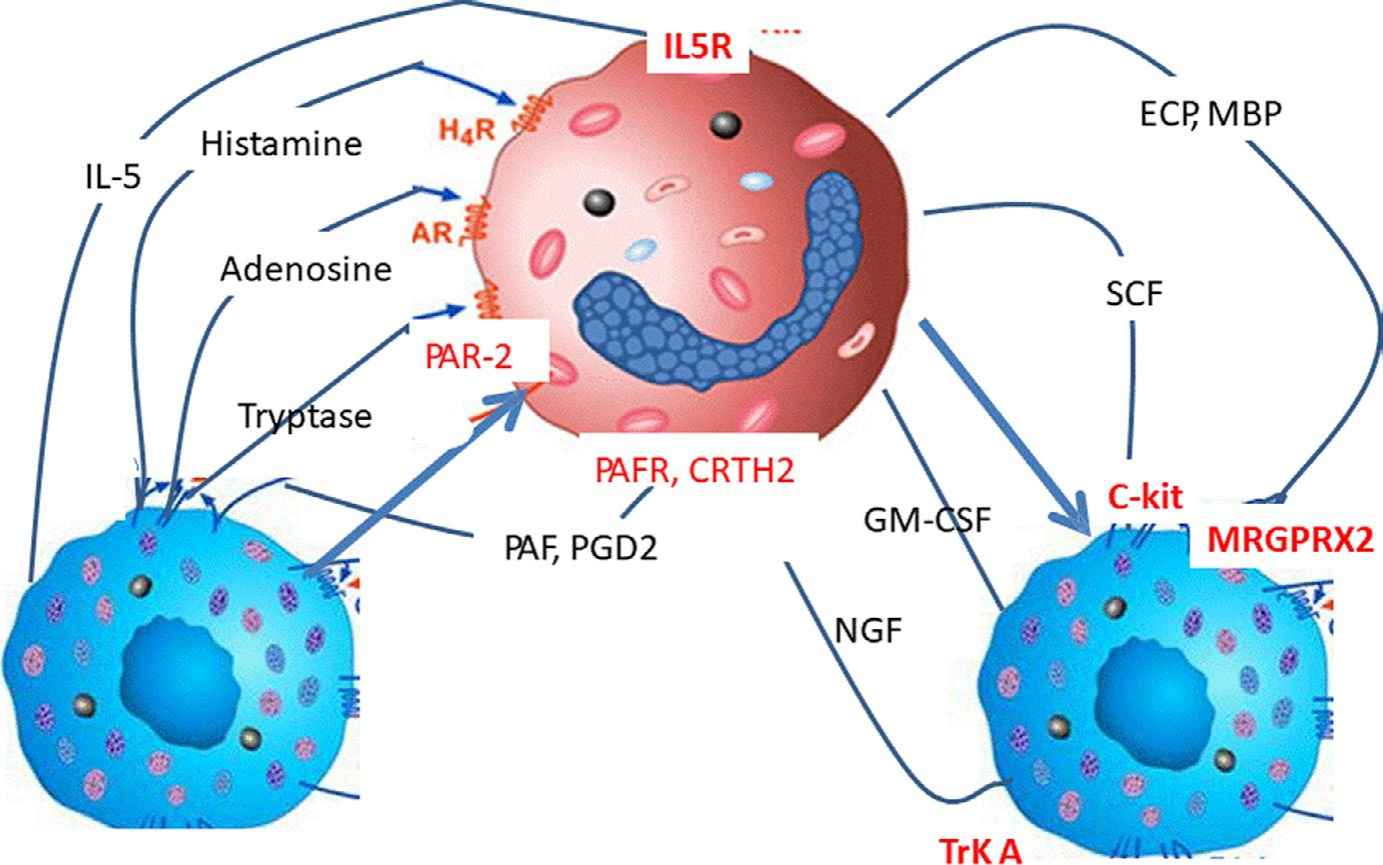
Examples of eosinophil‐mast cell interactions with effects on allergic inflammation

Eosinophils are often found together with mast cells (MCs), not only in allergy conditions, but also in other diseases, such as mastocytosis, systemic lupus erythematosus, bullous pemphigoid (BP), scleroderma, chronic graft‐versus‐host disease, cancers, atherosclerosis and idiopathic hypereosinophilic syndrome (HES). A type of pro‐inflammatory cross‐talk, called ‘Allergic Effector Unit’, with CD48 and 2B4 at the core, results in increased eosinophil chemotaxis, survival, degranulation, cytokine production and MC survival (Figure 2).^23^, ^24^, ^25^, ^26^, ^27^, ^28^ This cross‐talk also involves inhibitory receptors, that is, CD300a and Siglec‐7 or Siglec 8, on MCs and eosinophils.^25^ Specific monoclonal antibodies blocking CD48 and activating CD300a and Siglec‐7 or Siglec 8 could thus serve as potential drugs in future.

The activation of eosinophils results in several functional responses, including the release of granule proteins. However, control of the toxicity of these granule proteins within the eosinophil itself has not yet been identified. Recently, studies involving a combination of dye/immunostaining, immuno‐transmission electron microscopy and X‐ray micro‐crystallography have indicated that eosinophil major basic protein (MBP), a protein at the core of specific granules, is enclosed by a crystal lattice with amyloid‐like properties, enabling the inert storage of the otherwise toxic protein.^29^ Following eosinophil activation, MBP is mobilized, unpacked and released as non‐toxic oligomers. Upon secretion, an aggregation process is initiated, resulting in the generation of toxic MBP that is able to exert its microbiocidal effects. Interestingly, larger extracellular non‐toxic MBP aggregates can be detected in eosinophilic tissues, which may explain how tissue destruction by MBP can be limited. These data indicate that the toxicity of eosinophil MBP is controlled by functional amyloid formation.^29^

Granule proteins can also occur in the extracellular space, in association with so‐called ‘eosinophil extracellular traps’ (EETs).^30^ Eosinophil activation initiates the formation of EETs, consisting of catapult‐like release of mitochondrial DNA and granule proteins that form structures able to bind and kill bacteria. Thymic stromal lymphopoietin (TSLP) and *Staphylococcus aureus* serve as strong stimuli for EET generation in the context of eosinophil adhesion.^31^ Evidence that mitochondrial as well as nuclear DNA is released from eosinophils by an active process was obtained from live‐cell imaging analyses.^30^, ^31^, ^32^ As a component of the innate immune response, EETs were detected in several allergic, infectious and autoimmune eosinophilic diseases,^31^ including bronchial asthma, atopic dermatitis (AD),^33^, ^34^ eosinophilic esophagitis and chronic rhinosinusitis.^35^, ^36^

Recently, it has been proposed that eosinophil cytolysis is a manifestation of regulated necrosis, since the death process depends on receptor‐interacting protein kinase 3 (RIPK3) and mixed lineage kinase‐like (MLKL) protein.^37^ Notably, previous pharmacological activation of autophagy inhibits eosinophil cytolysis. Thus, suppressing the RIPK3‐MLKL pathway and stimulating autophagy in eosinophils may represent new therapeutic strategies to prevent eosinophil‐mediated tissue damage.

## 3 EOSINOPHILIC DISEASES

Eosinophilic inflammation occurs in various diseases,^38^ mostly allergic and parasitic diseases, but also in autoimmune diseases, in the course of malignancies (eg lymphoma), conditions induced by drugs (eg acetylsalicylic acid, cefoxitin, penicillin), and rare conditions, such as HES.^39^

### 3.1 Eosinophilic skin diseases

Eosinophilic inflammation is a characteristic of many skin diseases of allergic, infectious, haematologic, autoimmune or vascular origin (Table 1). There are some particular conditions in which eosinophilic inflammation is idiopathic, such as episodic angioedema,^40^ eosinophilic cellulitis (Well's syndrome), eosinophilic fasciitis, granuloma faciale, angiolymphoid hyperplasia with eosinophilia,^41^ eosinophilic folliculitis (Ofuji syndrome), as well as in the context of hypereosinophilia (HE) or HES.^42^, ^43^ IgG4‐related diseases and drug reactions with eosinophilia and systemic symptoms (DRESS) are multisystem diseases with strong eosinophilic inflammation in the skin.^44^ The first successful use of the anti‐IL‐5 monoclonal antibody mepolizumab was demonstrated in HES with skin involvement.^42^ AD, a type 2 cytokine‐driven inflammatory skin disease, is associated with increased eosinophil numbers both in blood and skin, and with elevated serum and urine levels of eosinophilic secretion products.^45^ A clinical trial investigated anti‐IL‐5, which target the major T‐cell‐derived eosinophilic growth and survival factor, for treatment of AD.^46^ In this small proof‐of‐concept trial, the primary study endpoint of clear improvement of AD was not reached, but some individual parameters showed promise. A recently published placebo‐controlled study indicated the efficacy of mepolizumab in patients with moderate to severe AD, demonstrating the improvement of clinical scores in patients treated with subcutaneously administered mepolizumab.^47^ Thus, future studies with anti‐IL‐5 or anti‐IL‐5R may yield additional positive results.

**TABLE 1:** Diseases involving eosinophilic inflammation of the skin

Type	Disease
Allergies	Eczema (atopic dermatitis)
	Urticaria, angioedema
	Drug reactions (eg, DRESS)
Infectious diseases	Helminth infestation
	Onchocerciasis
Autoimmune diseases	Bullous pemphigoid
	Pemphigus
	Eosinophilic fasciitis
	Eosinophilia myalgia syndrome
Vascular	Eosinophilic granulomatosis with polyangiitis (Churg‐Strauss syndrome)
Haematological Idiopathic	Hypereosinophilic syndromes (myeloid variant)
	Eosinophilic cellulitis (Well's syndrome)
	Episodic angioedema with eosinophilia (Gleich syndrome)
	Granuloma faciale
	Angiolymphomatoid hyperplasia with eosinophilia
	Hypereosinophilic syndrome

The role of eosinophils has been studied in bullous autoimmune dermatoses, such as pemphigus (involving auto‐antibodies against desmosomal proteins) and pemphigoid (involving auto‐antibodies against adhesion proteins).^48^ In the pre‐bullous stage of pemphigus, eosinophils infiltrate the epidermis, known as eosinophilic spongiosis. Desmoglein‐induced secretion of IL‐4, ‐5 and ‐13‐positive CD4 + T cells may attract eosinophils via IL‐5.^48^

In BP, which has a higher prevalence in the elderly population, blisters are filled with an eosinophil‐rich leukocyte infiltrate, with strongly enhanced activation of eosinophils, including those in the circulation.^49^ Eosinophils can also release mitochondrial DNA together with granule proteins (EETs) in proximity to apoptotic keratinocytes in BP patients.^34^, ^50^ Moreover, IL‐5‐activated eosinophils potentially contribute to blister formation by directly causing dermal‐epidermal separation.^50^ Eosinophils in BP also produce high levels of IL‐31,^51^ which may be involved in the strong itching sensation. Studies with the anti‐IL‐5 antibody mepolizumab have demonstrated efficacy for the treatment of hypereosinophilic diseases. The primary endpoint was the improvement of clinical symptoms, decrease in the blood eosinophil count and reduction of the prednisone dose to 10 mg or less per day for 8 or more consecutive weeks.^52^ Recently, a study indicated the efficacy of treatment with an anti‐eotaxin‐1 monoclonal antibody (bertilimumab) in BP and demonstrated an 81% decline in the subjects' BP Disease Area Index. The primary endpoint was safety, including the incidence of adverse effects.^53^, ^54^

### 3.2 Eosinophils in upper airway diseases

Chronic rhinosinusitis (CRS) is often considered an ‘eosinophilic’ upper airway inflammatory disease; however, there are several degrees of eosinophil involvement, recently described as non‐type 2, moderate type 2 and severe type 2 inflammation, ranging from CRS without to CRS with massive nasal polyps and comorbid asthma.^55^ The risk for and the severity of type 2 inflammation in CRS can vary considerably depending on the patient's place of residence (eg Asia vs. Europe) and may furthermore increase over time at places with low incidence.^56^ A possible role of *Staphylococcus aureus* has been discussed via release of enterotoxins SEA‐SEE and TSST‐1, or serine‐protease‐like proteins.^57^, ^58^ Serine‐protease‐like proteins can elicit IgE antibody responses in asthmatic patients and induce Th2 responses.^57^ Repeated intratracheal applications of SplD induced allergic asthma in C57BL/6 J mice and led to IL‐33 and eotaxin production, eosinophilia, bronchial hyperreactivity and goblet cell hyperplasia.^58^ Blocking IL‐33 activity with a soluble ST2 receptor consequently decreased lung eosinophils, innate lymphoid cells and T cells, as well as IL‐5 and IL‐13 production in lymph nodes, which resembled superantigen activities.^57^ Furthermore, in nasal polyp tissue samples, EET formation was observed.^36^ The understanding of eosinophil‐associated endotypes is likely to lead to new differentiated approaches in the management of CRS.^59^, ^60^

### 3.3 Eosinophils in lung diseases

In the clinical classification of eosinophilic lung disease, it is important to determine whether the disorder is limited to the lung or reflects a multisystemic disease. Lung‐limited eosinophilic diseases include eosinophilic pneumonia,^61^ parasite infection or eosinophilic airway diseases, such as asthma or allergic broncho‐pulmonary aspergillosis. Classical multisystem eosinophilic diseases include eosinophilic granulomatosis and polyangiitis (eGPA),^62^, ^63^ drug reactions with eosinophilia and HES. Eosinophils are increased in atopic as well as in non‐atopic asthma.^40^ In patients with asthma, treatment is guided by the absolute numbers of eosinophils in the blood,^64^ particularly in patients with severe eosinophilic asthma, where treatment with either IL‐5 or IL‐5Rc antibodies results in marked improvement,^65^, ^66^ even in systemic steroid‐dependent patients.^67^, ^68^ Interestingly, in other lung diseases, such as eGPA,^69^ anti‐IL‐5 treatment resulted in about 40% remission rates.^70^ Clinical studies with IL‐5 blockade are warranted for eosinophilic pneumonia or allergic broncho‐pulmonary aspergillosis.^69^, ^70^

### 3.4 Eosinophilic gastrointestinal diseases

Eosinophilic esophagitis (EoE) has increased in prevalence both in adults and in children since its first description in the 90s.^71^, ^72^ Diagnostic criteria include oesophageal stenosis with dysphagia and symptoms not otherwise explained, together with tissue eosinophilia (>15 eosinophils per high‐power field). The factors responsible for EoE range from environmental factors, including food allergens and the microbiome, interacting with the oesophageal epithelium with consecutive release of atopy‐enhancing cytokines, to genetic factors such as gender and genetic variants.^73^ A recent review revealed a delay of several years between symptom onset and diagnosis increasing the risk for fibrostenosis.^74^ Moreover, relatively high recurrence rates and low rates of spontaneous recovery were described, which indicate that EoE is a disorder persisting from childhood to adulthood.^74^

The majority of children with atopy have comorbid asthma, allergic rhinitis and/or atopic eczema (‘asthma of the oesophagus’). There is a broad overlap between eosinophilic esophagitis (EoE) and IgE‐mediated food allergies, particularly against cow's milk, beef, chicken, peanuts, chicken eggs, wheat and soy.^75^ Therefore, a critical evaluation of the relevance of specific IgE levels or skin tests against food proteins is crucial in the diagnostic work‐up, in addition to endoscopy with biopsy. The response to first‐line therapy with proton pump inhibitors (PPI) defines the new subtypes of eosinophilic GI diseases, namely PPI‐unresponsive and often IgE‐associated EoE versus PPI‐responsive oesophageal eosinophilia.^76^ However, it has been suggested that PPI responsiveness is not part of a diagnostic criteria but rather an effective treatment for some patients.^73^, ^77^, ^78^ Less is known about eosinophilic inflammation of other parts of the GI tract, such as eosinophil gastroenteritis, which are often associated with symptoms of irritable bowel syndrome.

### 3.5 Hypereosinophilia and hypereosinophilic syndrome

Over the past two decades, understanding of HE and HE‐induced organ damage has increased substantially.^79^, ^80^, ^81^ Neoplastic and reactive disorders underlying HE can be distinguished, and several proposals for the classification of HE‐related syndromes have been developed.^81^, ^82^, ^83^ Certain myeloid neoplasms and various reactive conditions are frequently associated with HE, but HE may also be seen with no apparent underlying disease (idiopathic HE).^82^, ^83^ In myeloid neoplasms, eosinophils are considered to belong to the malignant clone, whereas in reactive conditions, eosinophilia is usually a reactive process triggered by eosinophilopoietic cytokines. HE‐related organ damage, also known as HES, is seen in patients with haematologic malignancies, but also in reactive conditions.^83^ When no underlying disease is detected, the diagnosis is idiopathic HES.^83^ In patients with clonal HE (with or without HES), several different molecular markers can be detected, such as fusion genes involving PDGFRA (eg FIP1L1‐PDGFRA), PDGFRB, FGFR1, ABL1 or JAK2 (eg PCM1‐JAK2).^83^

## 4 MANAGEMENT AND NOVEL AND EXPERIMENTAL THERAPIES

Type‐2 inflammation is the predominant pattern in eosinophilic diseases. Eosinophils in blood and tissue serve as biomarkers.^68^, ^84^, ^85^, ^86^, ^87^ Detection of granular protein deposits in tissues is helpful. A value exceeding 1.5 × 10^9^/L that is unexplained by other diseases is regarded as HE, and when associated with organ damage or dysfunction in more than 2 organs is regarded as HES, or when associated with in one organ, as hypereosinophilic organ disease.^88^ Conventional therapy for HE and HES consists of anti‐inflammatory treatment using systemic glucocorticoids, immunosuppressives and UVA‐1 phototherapy,^35^, ^76^, ^78^, ^84^ as well as by eradicating underlying conditions. Pharmacotherapy often does not lead to satisfactory results, due to toxicity and lack of efficacy.

Clonal HE and clonal HES are treated with drugs acting at specific molecular targets, for example, FIP1L1‐PDGFRA (the most sensitive target of imatinib).^39^, ^83^, ^89^, ^90^, ^91^ For non‐responders or in those developing acute leukaemia, conventional chemotherapy and hematopoietic stem cell transplantation have to be considered, while in irreversible endomyocardial fibrosis and related heart failure, heart transplantation remains an option.^92^ Biologics with specificity for eosinophils, targeting IL‐5 and IL‐5R, such as mepolizumab, reslizumab or benralizumab have shown promising effects.^42^, ^52^, ^93^

Alternative approaches for targeting type‐2 inflammation consist of substances affecting eosinophil chemotaxis, such as anti‐eotaxin‐1 antibodies (bertilimumab), which have recently demonstrated promising results,^53^ as well as substances that block production of the transcription factor GATA‐3, by means of a GATA‐3 mRNA‐specific antisense molecule, HgD40. HgD40 belongs to the class of DNAzymes, which are synthetic DNA molecules. HgD40 contains a catalytic domain through which it exerts its cleavage function once sequence‐specific domains bind to the GATA‐3 mRNA. Clinical efficacy of this treatment by inhalation has been shown following allergen provocation in mild and moderate asthmatic patients.^94^ Clinical responses are associated with a significant reduction of eosinophils in the sputum. These observations have been now extended to a subgroup of chronic obstructive pulmonary disease patients, characterized by the presence of at least 2% sputum eosinophils.^95^

## 5 CONCLUSIONS

Eosinophils are evolutionarily present from the early vertebrates.^96^ They contribute to marked inflammation in tissues with both protective (eg against parasites) and pathogenic effects (eg hypereosinophilic conditions). In terms of general health effects, transgenic mouse models have shown that it is possible to live without eosinophils, but that the organism is better off having them. Eosinophilic diseases manifest in various organs, most notably in the skin, the GI tract, the airways, the heart, and with rare involvement of the nervous system and the vasculature. HES involves symptoms in various organs, with a fusion gene mutation or reactive IL‐5 up‐regulation. To manage hypereosinophilic conditions, history, physical examination, laboratory investigations, histology and imaging analyses are performed. Genetic analyses for mutations allow distinction between myeloid and lymphoid variants.

While systemic glucocorticoids remain the mainstay of treatment, particularly in the initial phase, prognosis has improved markedly through the introduction of targeted therapies implementing anti‐IL‐5 antibodies and kinase inhibitors.^43^, ^97^ Through a better understanding of eosinophil function in health and disease, and with the development of specific kinase inhibitors as well as targeted biologics, the era of precision medicine has now arrived in the field of eosinophilic diseases. Recent findings in transgenic mice and preliminary human studies targeting new receptors on eosinophils and blockade of transcription factors impart hope for further progress in the management of eosinophilic diseases.

## Data Availability

n/a
